# Outcome of Ultrasound-Guided Versus Fluoroscopy-Guided Versus Cephalic Cutdown Permanent Pacemaker Implantation

**DOI:** 10.7759/cureus.100698

**Published:** 2026-01-03

**Authors:** Jiwan Pradhan, Yusif Shakanti, Victoria M Robinson, Hamidreza Abbasi, Cathy Holt, Nadim Malik

**Affiliations:** 1 Cardiology, Stockport NHS Foundation Trust, Stockport, GBR; 2 Cardiology, Faculty of Biology, Medicine and Health, University of Manchester, Manchester, GBR

**Keywords:** cardiac arrhythmia, cephalic vein cutdown, complications to pacemaker implantation, fluoroscopy-guided access, permanent pacemaker implantation (ppm), ultrasound-guided access

## Abstract

Introduction: The universal standard practice for central venous access during permanent pacemaker (PPM) implantation is fluoroscopy with venogram. Ultrasound-guided access is increasingly encouraged to minimize radiation, contrast load, and inadvertent injury to surrounding structures. However, its complication profile in PPM implantation remains uncertain. This study compared complications and radiation exposure across ultrasound-guided, fluoroscopy-guided, and cephalic vein cutdown approaches.

Methods: A retrospective cross-sectional study of 202 consecutive patients undergoing elective or urgent PPM implantation by experienced operators at a single center (August 2021-August 2022) was performed. Access-related complications, fluoroscopy time, and radiation dose were analyzed. Complications were predefined as one or more of the following events: arterial puncture, haematoma, pneumothorax, lead or device failure, or procedure failure.

Results: Among the cohort, 56/202 (28%) underwent ultrasound-guided access, 114/202 (56%) fluoroscopy-guided access, and 32/202 (16%) cephalic cutdown. Complication rates were 5.4% (3/56) for ultrasound-guided, 9.6% (11/114) for fluoroscopy-guided, and 9.4% (3/32) for cephalic cutdown (χ² test, p = 0.62). Radiation dose differed significantly between groups (Kruskal-Wallis test, p < 0.001), with the lowest median (IQR) exposure in the cephalic cutdown group at 10.1 mGy (6.7-17.0), followed by ultrasound-guided access at 13.4 mGy (9.2-23.1) and fluoroscopy-guided procedures at 19.2 mGy (12.2-31.0).

Conclusion: Ultrasound-guided access was associated with the lowest observed complication rate, although this was not statistically significant, likely reflecting limited sample size. Radiation exposure varied significantly by access method, with ultrasound guidance offering an intermediate profile between cephalic cutdown and fluoroscopy. These findings support the safety of ultrasound-guided access and highlight the need for larger studies to confirm its role in reducing complications and radiation burden in PPM implantation.

## Introduction

Gaining central venous access is a fundamental initial step in implanting transvenous permanent pacemakers (PPM). Complications relating to accessing central venous channels impact the eventual outcome of PPM procedures. In the literature, the majority of PPM complications relate to accessing central venous channels [1]. Standard clinical practice involves access through the subclavian or axillary vein using fluoroscopy with venogram imaging or through cephalic vein cutdown. In current practice, current guidelines recommend either approach as suitable [2].

Ultrasound imaging is increasingly used to gain central venous access, particularly in intensive care and emergency medicine settings [3]. Ultrasound-guided PPM implantation is also described as a helpful technique for achieving a safe and uncomplicated puncture [4]. Ultrasound imaging allows direct visualization of the vessel and tracks the puncture needle's approach, theoretically reducing the risk of access-related complications [5]. Ultrasound imaging for central venous access also avoids radiation exposure during the initial stage; however, fluoroscopy is still required for the remainder of the PPM implantation procedure [6].

Several recent trials have compared vascular access techniques for PPM implantation. A randomized trial demonstrated that ultrasound-guided axillary access reduced fluoroscopy exposure compared with the standard fluoroscopic technique, while another trial showed intra-pocket ultrasound-guided axillary puncture as a safe alternative to cephalic cutdown [7,8]. Larger multicenter studies are still needed to confirm these findings.

This observational study aimed to retrospectively review data from consecutive patients undergoing emergency or elective PPM implantation at a single center over a 12-month period, comparing complication rates associated with ultrasound-guided versus fluoroscopy-guided vascular access and cephalic cutdown. Specifically, we examined immediate and 30-day access-related complications, as well as cumulative radiation dose during the entirety of the procedure.

## Materials and methods

We conducted a retrospective cross-sectional study at a medium-sized district general hospital in the United Kingdom. The study period spanned from August 2021 to August 2022.

All consecutive adult patients (≥18 years) who underwent PPM implantation during the study period were eligible for inclusion. Patients undergoing lead revision were excluded. In cases where the initial vascular access method was unsuccessful and an alternative technique was employed, only the initial method was considered for the analysis of access-related complications.

The primary aim was to identify the rate of access-related complications, observed either intra-procedurally or within 30 days of the procedure, for each type of vascular access. Late venous complications were excluded due to their multifactorial nature. Complications were predefined as one or more of the following events: arterial puncture, hematoma, pneumothorax, lead or device failure, or procedure failure; they were collected as a categorical variable [7,8]. The secondary aim was to compare radiation dose (mGy) and total fluoroscopy time (minutes), which were collected as continuous variables. Additional variables collected included patient demographics (age, sex), comorbidities (e.g. hypertension, diabetes, or asthma), and anticoagulation/antiplatelet status where available.

Patient allocation to access technique was determined by procedural scheduling rather than patient characteristics or urgency of the procedure, as operators staffed the catheterization laboratory on different days and used their standard access approach. This minimized systematic selection bias, although residual confounding cannot be excluded due to the observational study design.

The primary exposure variable was the method of vascular access, categorized as ultrasound-guided venous access, fluoroscopy-guided access (axillary or subclavian vein), or cephalic vein cutdown.

Data were collected retrospectively from operative reports, electronic medical records, and radiological documentation during the index admission and subsequent follow-up within the same hospital system. As this was a retrospective study, delayed or externally managed complications may have been undercaptured. Radiation data were extracted from procedural logs in the hospital’s imaging system. Complication events were identified through procedural documentation, post-procedural imaging, and clinical follow-up. Complications were adjudicated independently by an experienced operator. Two independent reviewers extracted data using a standardized data collection template based on predefined criteria.

Baseline characteristics were compared between access groups using one-way ANOVA for continuous variables and chi-squared tests for categorical variables (Fisher’s exact test was applied where expected cell counts were <5). Complication rates by access method were compared using the chi-squared test. Subgroup analyses of complication rates according to antiplatelet use, comorbidity status, and direct oral anticoagulant (DOAC) therapy were performed using Fisher’s exact test. For radiation exposure (fluoroscopy time and radiation dose), between-group differences were assessed using the Kruskal-Wallis test, with Dunn’s post-hoc test applied for pairwise comparisons. A two-tailed p-value <0.05 was considered statistically significant. Missing data were handled by complete case analysis, with no imputation performed. Missing data were infrequent and did not appear to be systematically associated with a specific access group; however, we acknowledge that complete case analysis may introduce bias if missingness was not completely at random, and this represents a limitation of the study.

Analyses were conducted using univariable methods; as multivariable adjustment was not performed, residual confounding cannot be excluded.

The sample size was determined by the number of eligible patients during the study period. As this was an exploratory analysis, no formal sample size calculation was performed. Selection bias was minimized by including all consecutive eligible patients. Observer bias was reduced through the use of standardized definitions.

## Results

A total of 202 patients underwent PPM implantation during the study period. Of these, 56 (28%) received ultrasound-guided axillary venous access, 114 (56%) underwent fluoroscopy-guided axillary or subclavian access, and 32 (16%) underwent cephalic vein cutdown. The median age of the cohort was 81 years (IQR: 73-87), and 67% were male.

Comorbidity data were available for 188 patients; 14 out-of-area patients lacked comprehensive past medical history records. Demographic and comorbidity data were unavailable for these patients, but they were retained in the overall cohort for complication analysis. Among the 188 patients with available data, 135 (72%) had one or more comorbidities, and 53 (28%) had none. The most prevalent comorbid conditions were hypertension, diabetes mellitus, chronic kidney disease (CKD), peripheral vascular disease (PVD), and chronic obstructive pulmonary disease (COPD)/asthma. DOAC use was documented in 5%, and antiplatelet therapy in 17% of the cohort. A total of 70 patients (35%) underwent their procedure electively. The complete breakdown of variables is presented in the supplementary table.

Patients across the three venous access groups were broadly comparable in age, sex distribution, and most comorbidities. Hypertension was more prevalent in the ultrasound-guided group compared with the fluoroscopy-guided and cephalic cutdown groups (68% vs 51% and 38%, p = 0.02). Trends toward higher rates of diabetes and COPD/asthma were noted in the ultrasound group, though these did not reach statistical significance. The use of antiplatelet or anticoagulant therapy and the proportion of elective procedures were similar across all groups. A detailed breakdown of baseline characteristics by access method is provided in Table 1.

**Table 1:** Baseline demographic and clinical characteristics of patients undergoing permanent pacemaker implantation, stratified by vascular access method Baseline demographic and clinical characteristics of patients undergoing permanent pacemaker implantation, stratified by vascular access technique. Age is presented as mean ± standard deviation (SD). Categorical variables are presented as n (%). p-values are derived from one-way ANOVA for age and Pearson’s chi-square test of independence for categorical variables. For each chi-square test, the null hypothesis was that the distribution of the characteristic is the same across ultrasound-guided, fluoroscopy-guided, and cephalic approaches. Bold p-values indicate statistical significance (p < 0.05). Abbreviations: SD – standard deviation; DOAC – direct oral anticoagulant; COPD – chronic obstructive pulmonary disease Hypertension was the only baseline characteristic that differed significantly between groups, being more prevalent in the ultrasound-guided cohort compared with fluoroscopy and cephalic approaches (χ² = 7.3, p = 0.02).

Characteristic	Ultrasound-guided (n=56)	Fluoroscopy-guided (n=114)	Cephalic cutdown (n=32)	Test statistic	p-value
Age	81 ± 7	79 ± 11	82 ± 8	F=2.1	0.13
Male	37 (66%)	79 (69%)	20 (63%)	χ²=0.58	0.75
Hypertension	36 (68%)	55 (51%)	11 (38%)	χ²=7.34	0.02
Diabetes	14 (26%)	21 (18%)	2 (7%)	χ²=4.52	0.10
Chronic kidney disease	13 (25%)	25 (22%)	5 (17%)	χ²=0.68	0.71
Peripheral vascular disease	2 (4%)	3 (3%)	0 (0%)	χ²=1.06	0.59
COPD/Asthma	8 (16%)	18 (16%)	1 (3%)	χ²=3.59	0.17
DOAC use	5 (9%)	4 (4%)	1 (3%)	χ²=2.43	0.30
Antiplatelet use	12 (21%)	20 (18%)	3 (9%)	χ²=2.08	0.35
Elective patients	19 (34%)	41 (36%)	10 (31%)	χ²=0.26	0.88

Procedural outcomes and complications

The overall complication rate was 8.4% (17/202), with pneumothorax (7/202, 3.5%) and lead-related complications (5/202, 2.5%) being the most frequent events. These findings are consistent with previously reported complication rates of approximately 5-10% following PPM implantation in large registries [9-11]. In our cohort, pneumothorax occurred in 3.4% of cases, slightly above the reported incidence of 1-3%; lead-related complications were observed in 2.5%, lower than the published range of 2-5%; and hemorrhagic events occurred in 1%, comparable with reported rates of 1-5% [9-11]. Taken together, these data suggest that procedural safety outcomes at our center align with internationally reported averages.

When stratified by venous access technique, complications occurred in 3/56 (5.4%) of ultrasound-guided axillary cases, 11/114 (9.6%) of fluoroscopy-guided cases, and 3/32 (9.4%) of cephalic cutdown cases (Table 2). There was no statistically significant difference in complication rates between the three groups (χ² test, p = 0.62). By individual event type, pneumothorax occurred most frequently in the fluoroscopy-guided group (5/114, 3.6%), while lead-related complications were more common in the cephalic cutdown group (2/32, 6.2%).

**Table 2:** Procedural complications stratified by the venous access method The overall incidence of access-related complications was 8.4% (17/202). Total complication rates did not differ significantly between ultrasound-guided axillary vein puncture (5.4%), fluoroscopy-guided axillary vein puncture (9.6%), and cephalic cutdown (9.4%) approaches (χ² = 0.94, p = 0.62). For the chi-squared test, the null hypothesis was that the proportion of patients experiencing complications was the same across all three access methods. Individual complication types, including pneumothorax, hemorrhagic events, procedure failure, and device/lead failure, are presented descriptively; statistical comparison was not performed for these subgroups due to low event counts as this would be statistically misleading.

Complication	Ultrasound	Fluoroscopy	Cephalic	Test	p-value
Pneumothorax	2 (3.6%)	5 (4.4%)	0 (0.0%)	-	-
Hemorrhagic	0 (0.0%)	2 (1.8%)	0 (0.0%)	-	-
Procedure failure	1 (1.8%)	0 (0.0%)	1 (3.1%)		-
Device/lead failure	0 (0.0%)	3 (2.6%)	2 (6.2%)	-	-
Device/lead infection	0 (0.0%)	1 (0.9%)	0 (0.0%)	-	-
Total number of complications	3 (5.4%)	11 (9.6%)	3 (9.4%)	χ²=0.94	0.62

Subgroup analysis by antiplatelet use and comorbidity status showed no significant differences in complication rates. Patients receiving antiplatelet therapy had 2/35 complications (5.7%) compared with 15/162 (9.3%) in those not on antiplatelets (Fisher’s exact test, p = 0.74). Similarly, complication rates did not differ significantly between patients with and without comorbidities (11.3% vs 8.1%; Fisher’s exact test, OR 0.69, p = 0.57). Although no complications were observed among the 10 patients on DOAC therapy, the limited sample size precludes firm conclusions.

Radiation exposure

Radiation dose and fluoroscopy time data were available for the majority of patients. Radiation dose data were missing in 32/202 patients (15.8%), with comparable proportions across ultrasound-guided (8.9%), fluoroscopy-guided (17.5%), and cephalic cutdown (21.9%) cases, suggesting a low risk of systematic bias. Fluoroscopy time data were missing in 30/202 patients (14.9%), including five ultrasound-guided, 19 fluoroscopy-guided, and six cephalic cutdown cases. The complete dataset is provided in the supplementary table.

Both measures differed significantly between the three venous access groups (Kruskal-Wallis test, p < 0.001). Median fluoroscopy time was lowest with cephalic vein cutdown (2.1 minutes), intermediate with ultrasound-guided access (2.4 minutes), and highest with fluoroscopy-guided access (3.5 minutes). Post-hoc analysis using Dunn’s method confirmed that fluoroscopy-guided procedures required significantly longer screening time compared with both ultrasound-guided and cephalic cutdown approaches (p < 0.001 for both).

Radiation dose showed a similar trend, with the lowest median exposure observed in cephalic cutdown cases (10.1 mGy), followed by ultrasound-guided (13.4 mGy) and fluoroscopy-guided access (19.2 mGy) (Table 3).

**Table 3:** Radiation dose and fluoroscopy time stratified by the venous access method Data available for 170 patients. Fluoroscopy time and radiation dose are presented as median with interquartile range (IQR). The Kruskal–Wallis test was used for overall comparison, with the null hypothesis that the distribution of radiation exposure is the same across all three vascular access methods. Post-hoc analysis with Dunn’s test confirmed that fluoroscopy-guided access had significantly longer fluoroscopy time and higher radiation dose compared with both ultrasound-guided and cephalic cutdown approaches (p < 0.001).

Variable	Ultrasound	Fluoroscopy	Cephalic	Test	p-value
Fluoroscopy time (mins)	2.4 (1.5-3.3)	3.5 (2.4-5.7)	2.1 (1.3-2.6)	H=25.8	<0.001
Radiation dose (mGy)	13.4 (9.2-23.1)	19.2 (12.2-31.0)	10.1 (6.7-17.0)	H=16.6	<0.001

## Discussion

Ultrasound-guided access in our study was associated with the lowest observed complication rate among the three approaches, supporting its favorable safety profile. Although this difference did not reach statistical significance, the numerical trend may reflect limited sample size and lack of power. Differences in baseline hypertension are unlikely to account for the observed variation in access-related complication rates, as hypertension is not a recognized independent risk factor for such complications. Radiation exposure with ultrasound was not as low as with cephalic cutdown but was significantly lower than with fluoroscopy-guided access, underscoring its potential to reduce both operator and patient exposure.

Ultrasound guidance also offers real-time vessel visualization, which, in theory, can help prevent inadvertent arterial puncture and pneumothorax, two of the most concerning complications of blind or fluoroscopic access [12]. These findings are in keeping with broader literature where ultrasound-guided vascular access has consistently been shown to reduce complications across specialties, including critical care, anesthesia, and interventional cardiology [1,3,13,14]. Despite this, its adoption in device implantation has lagged. Barriers may include limited availability of trainers and the learning curve associated with mastering the technique, though once established, ultrasound guidance offers reproducible and safe access.

A key strength of this study is that it represents real-world, consecutive cases of permanent pacemaker implantation at a single center, providing a pragmatic picture of current practice. All three commonly used venous access techniques were included, allowing direct comparison within the same cohort. Importantly, both fluoroscopy time and radiation dose were systematically recorded and analysed, which are often incompletely reported in the literature. The inclusion of predefined complication categories and subgroup analyses by comorbidity and medication status further strengthens the reliability of the findings.

Limitations 

The present observational study was a small, retrospective analysis conducted at a single center. While the findings are consistent with previous smaller studies, they also highlight the need for larger, multicenter randomised controlled trials to confirm the safety and efficacy of ultrasound guidance for PPM implantation [7,8]. The study design inevitably introduces limitations, including potential selection bias and limited generalizability. The sample size, although reflective of local practice, may have been underpowered to detect statistically significant differences in uncommon complications.

Within the fluoroscopy-guided group, both axillary and subclavian venous access were used, which may have introduced additional heterogeneity that could not be explored due to limited power.

Procedure complexity and device type (dual- versus single-chamber implantation), which may influence fluoroscopy time and radiation dose, were not specifically adjusted for and could have contributed to variability between groups. In addition, image acquisition settings were not fully standardized across operators, which may have contributed to variability in radiation dose.

Fourteen ‘treat-and-return’ patients lacked complete comorbidity data, although they were retained in the complication analysis to avoid underestimating event rates.

Finally, operator experience, which is known to influence procedural success and complication rates, including pneumothorax, was not fully captured and may have contributed to variability between groups rather than the access method alone.

Clinical implications

Despite these limitations, our findings highlight the relative safety of ultrasound-guided access and its potential to reduce radiation exposure compared with fluoroscopy-guided techniques [8]. Cephalic cutdown remains a valuable option for minimising radiation, although its technical feasibility may be limited by anatomical variation and operator experience [11]. Given that ultrasound guidance is already widely established in other procedural settings, greater adoption in device implantation could enhance patient and operator safety [15]. Incorporating structured ultrasound training into device implantation practice has the potential to improve procedural safety by reducing radiation exposure. Although complication rates appear favorable, validation in larger studies is required. With further evidence, ultrasound guidance has the potential to become a widely accepted default training strategy for venous access in pacemaker implantation. Potential barriers to implementation include resource availability, staffing constraints, and variability in training.

Absolute radiation doses were well below thresholds for deterministic injury [16]. The lower radiation exposure observed with cephalic cutdown and ultrasound-guided access may be attributable, in part, to differences in access technique. The reduction in median exposure may be clinically meaningful when considering cumulative radiation exposure over time, particularly for staff performing high procedural volumes; however, this was not the primary focus of the present study and warrants further investigation.

## Conclusions

This real-world, retrospective study from a medium-volume center compared outcomes of ultrasound-guided access, fluoroscopy-guided venous access, and cephalic cutdown for PPM implantation. While no statistically significant differences in complication rates were observed, radiation exposure differed significantly between techniques. We acknowledge that baseline imbalances and limited statistical power constrain the strength of safety inferences.

In keeping with prior reports, our findings suggest that ultrasound-guided venous access is a feasible and safe approach in routine practice. However, conclusions regarding comparative effectiveness from this study should be interpreted cautiously. We believe that larger, adequately powered randomized controlled trials are required to more definitively assess the impact of ultrasound-guided access on patient safety, radiation exposure, and cost-effectiveness relative to fluoroscopy-guided implantation and cephalic cutdown.

## Appendices

Table 4 presents the raw or unfiltered dataset.

**Table 4:** Raw/unfiltered dataset (Supplementary Material) Patient-level data, including DOB, sex, elective status, anticoagulant/antiplatelet use (DOAC, antiplatelets), comorbidities (HTN, diabetes, CKD, PVD, COPD/asthma), device type, implant indication, venous access approach, procedural complications, fluoroscopy time (mins), radiation dose (mGy), and out-of-area status. “na” denotes data not available. Binary fields (e.g., DOAC, Antiplatelets, HTN, Diabetes, CKD, PVD, COPD/Asthma, Elective, Out of area patient): 1 = Present/Yes; 0 = Absent/No. Key: Approach: 1 = Ultrasound, 2 = Fluoroscopy, 3 = Cephalic Complications: 0 = No complications, 1 = Pneumothorax, 2 = Hemorrhagic complications, 3 = Failure of procedure, 4 = Lead failure/displacement, 5 = Device/lead infection, 6 = Patient died within 30 days Abbreviations: DOAC = Direct Oral Anticoagulant; HTN = Hypertension; DDD = Dual Chamber Pacemaker; VVI = Single Chamber Ventricular Pacemaker; DOB = Date of Birth; CKD = Chronic Kidney Disease; PVD = Peripheral Vascular Disease; COPD = Chronic Obstructive Pulmonary Disease; CRT-P = Cardiac Resynchronization Therapy Pacemaker; CRT-D = Cardiac Resynchronization Therapy with Defibrillator; ICD = Implantable Cardioverter Defibrillator; mGy = milligray (unit of radiation dose)

DOB	SEX	Elective	DOAC	Antiplatelets	HTN	Diabetes	CKD	PVD	COPD/Asthma	Device	Indication	Approach	Complications	Fluoroscopy time (mins)	Radiation Dose (mGy)	Out of area patient
16/07/1952	M	0	0	1	0	1	0	1	0	DDD	second degree heart block	1	0	3.19	21.8	0
20/09/1932	F	1	0	0	1	0	1	0	0	DDD	tachy brady syndrome	1	0	2.15	8.48	0
06/01/1950	F	0	0	1	na	na	na	na	na	DDD	Complete Heart Block	1	0	2.41	8.13	1
18/06/1945	M	0	0	0	1	0	0	0	0	VVI	tachy brady syndrome	1	0	2.41	16.6	0
27/10/1934	M	0	1	0	1	1	0	0	0	DDD	tachy brady syndrome	1	0	3.21	13.4	0
22/12/1941	M	0	0	0	1	1	0	0	1	DDD	second degree heart block	1	0	1.15	15.8	0
20/07/1934	M	1	0	0	1	0	0	0	0	VVI	atrial fibrillation and symptomatic pauses	1	0	2.45	30.8	0
07/09/1933	M	0	0	0	1	0	0	0	0	DDD	tachy brady syndrome	1	3	37	406	0
08/04/1938	M	0	0	1	1	0	0	0	0	DDD	severe bradycardia	1	0	2.37	11.8	0
21/01/40	M	1	1	0	0	1	0	0	1	DDD	VVI pacemaker upgraded to DDD pacemaker.	1	0	1.12	8.21	0
02/10/1944	F	0	0	0	1	1	1	0	1	DDD	Complete Heart block	1	0	2.45	17.1	0
07/02/1937	M	1	0	0	1	0	0	0	0	VVI	atrial fibrillaiton with pauses	1	0	2.25	19	0
15/03/1934	F	0	0	0	1	0	1	0	0	DDD	tachy-brady syndrome with >6 second pause	1	0	2.3	13	0
27/10/1930	F	0	0	1	1	1	0	0	0	VVI	atrial fibrillation and pauses	1	0	3.21	7.49	0
26/03/1944	F	0	0	0	1	0	0	0	0	VVI	pauses up to seven seconds	1	0	2.03	13.2	0
15/09/1935	F	1	0	0	0	1	0	0	1	DDD	Pre-erosion on the inferior segments	1	0	1.32	2.11	0
27/10/1937	F	1	0	1	1	0	1	0	1	DDD	complete heart block	1	0	2.01	9.96	0
07/11/1948	M	1	0	1	1	0	0	0	0	DDD	trifasicular block	1	0	4.44	43.1	0
03/03/1940	M	0	1	0	1	0	0	0	0	VVI	tachy brady syndrome	1	0	0.52	4.95	0
09/05/1952	M	1	0	0	1	1	0	0	0	DDD	pauses up greater than 6 seconds	1	0	1.47	8.27	0
03/12/1958	M	0	0	0	1	0	0	0	0	DDD	complete heart block	1	0	1.34	9.53	0
16/06/1946	F	0	1	0	0	0	0	0	0	DDD	complete heart block	1	0	3.36	24.4	0
07/10/1936	M	0	1	0	1	0	0	0	0	DDD	pauses up greater than 6 seconds	1	0	4.17	26.4	0
05/06/1937	F	0	0	0	1	0	0	0	0	DDD	complete heart block	1	1	4.29	12.9	0
08/02/1937	M	1	0	1	1	0	1	0	0	DDD	trifasicular block	1	0	1.48	17.7	0
23/07/1936	M	0	0	1	0	0	1	0	0	DDD	trifasicular block	1	0	na	na	0
28/10/1935	M	0	0	0	0	1	1	0	0	DDD	sinus bradycardia	1	0	na	na	0
01/15/1930	M	0	0	0	1	0	0	0	0	DDD	tachy brady syndrome	1	0	na	na	0
16/02/1937	M	0	0	1	1	0	1	1	0	VVI	second degree heart block	1	0	na	na	0
07/03/1948	M	0	0	0	0	0	0	0	0	VVI	second degree heart block	1	0	na	na	0
04/10/1937	M	0	0	0	0	0	0	0	0	DDD	p wave asystole	1	0	1.09	8.93	0
15/04/1937	M	0	0	1	0	1	1	0	0	VVI	pauses 4 seconds	1	0	1.49	8.87	0
26/08/1951	M	0	0	0	0	0	0	0	0	DDD	tachy brady syndrome	1	0	2.03	6.77	0
04/04/1950	M	1	0	0	1	0	0	0	0	DDD	tachy brady syndrome	1	0	1.03	8.27	0
29/04/1947	M	0	0	0	0	0	0	0	0	DDD	pauses	1	0	3.18	25.2	0
12/10/1945	M	0	0	1	1	1	0	0	0	DDD	second degree heart block	1	0	4.55	71.8	0
07/12/1950	F	1	0	0	1	0	1	0	1	DDD	complete heart block	1	0	4.37	15	0
10/04/1943	F	0	0	0	1	0	1	0	0	DDD	complete heart block	1	0	13.37	111	0
02/11/1931	M	1	0	0	0	0	0	0	0	VVI	Slow AF	1	0	1.09	10	0
17/03/1935	F	1	0	1	1	0	0	0	0	DDD	PAF, brady	1	0	5.29	23	0
12/10/1951	M	0	0	0	1	1	0	0	0	VVI	Tachy-brady	1	0	1.07	9.81	0
13/02/1944	M	0	0	0	na	na	na	na	na	DDD	Sinus pause 10sec	1	0	3.16	24	1
09/06/1938	M	0	0	0	0	0	1	0	1	DDD	Tachy-brady	1	0	2.02	16.6	0
12/05/1950	M	1	0	0	1	0	0	0	0	DDD	PAF with pauses 10sec	1	0	2.55	18.7	0
27/04/1930	M	0	0	0	1	1	0	0	0	DDD	CHB, RBBB	1	0	1.49	8.81	0
28/06/1935	M	0	0	0	0	0	0	0	1	VVI	CHB	1	0	1.38	6.11	0
18/09/1935	F	0	0	0	1	0	0	0	0	VVI	2:1 AV blcok	1	0	3.36	18.8	0
30/07/1948	M	1	0	0	0	1	0	0	0	DDD	AF with pauses	1	0	1.24	11	0
19/01/1933	F	1	0	0	1	0	0	0	0	DDD	Trifasciciular block	1	0	3.1	12	0
02/03/1940	M	0	0	0	1	0	0	0	0	DDD	Mobitz 2	1	0	6.31	46.7	0
09/04/1946	M	1	0	0	1	0	0	0	0	DDD	Mobitz 2	1	0	3.25	23.1	0
29/11/1934	M	1	0	0	1	0	0	0	0	DDD	AF with pauses	1	0	3.09	23.6	0
20/04/1949	F	0	0	0	na	na	na	na	na	DDD	CHB	1	0	3.44	11.4	1
28/12/1946	F	0	0	0	1	0	0	0	0	DDD	na	1	0	5.19	27.5	0
21/08/1942	F	0	0	0	0	0	0	0	0	DDD	CHB	1	0	2.1	15.5	0
19/06/1934	F	1	0	0	0	0	1	0	0	DDD	Tachy/brady syndrome	1	1	2.38	12	0
16/3/46	M	1	0	1	0	0	0	0	0	DDD	Complete Heart Block	2	0	3.22	20.1	0
31/03/1930	M	1	0	1	1	1	1	0	0	DDD	Complete Heart Block	2	0	2.47	21.5	0
24/09/1934	F	0	1	1	1	0	1	1	0	DDD	tachy brady syndrome	2	0	1.46	7.66	0
07/11/1927	F	1	0	0	1	1	0	0	0	VVI	tachy brady syndrome	2	0	2.37	15.1	0
17/3/30	M	0	0	0	1	0	1	1	1	DDD	Complete Heart Block; patient relatively asymptomatic	2	0	3.1	13.6	0
26/01/1937	M	0	na	na	na	na	na	na	na	DDD	Complete Heart Block	2	0	5.55	29.1	1
14/09/1955	M	0	na	na	na	na	na	na	na	DDD	tachy brady syndrome	2	0	2.34	19.4	1
24/03/1942	F	0	0	0	1	0	0	0	1	DDD	complete heart block	2	0	4	28.4	0
21/10/1939	M	1	0	1	1	0	0	0	0	DDD	First degree AV block with intermittent bradycardia	2	4	7.46	52	0
04/05/1943	F	0	0	0	0	0	0	0	0	VVI	Symptomatic slow atrial fibrillation	2	0	2.13	9.78	0
21/12/1939	M	0	0	0	0	0	0	0	0	DDD	second degree heart block	2	0	5.09	31.1	0
02/01/1933	F	0	1	0	0	0	0	0	1	VVI	Slow atrial fibrillation	2	0	3.06	9	0
02/10/1954	M	0	0	0	1	0	0	0	1	DDD	Sinus Bradycardia	2	0	9.15	42.8	0
22/09/1935	M	1	0	0	0	0	0	0	0	VVI	atrial fibrillation and pauses	2	0	10.48	69.9	0
10/12/1951	M	0	0	0	1	0	0	0	0	DDD	second degree heart block	2	0	9.38	73.7	0
23/09/1954	M	0	0	0	0	0	0	0	0	DDD	severe bradycardia	2	1	6.57	24.2	0
05/05/1934	M	0	0	0	0	0	0	0	0	VVI	complete heart block	2	0	4.43	40.8	0
13/04/1948	M	1	0	1	0	0	0	0	0	DDD	complete heart block	2	0	5.66	22.5	0
23/02/1953	M	1	0	1	1	1	0	0	0	DDD	second degree heart block	2	0	7.13	29.7	0
21/07/1927	F	0	0	0	0	1	0	0	0	VVI	complete heart block	2	0	1.33	7.46	0
15/09/1936	M	1	0	0	1	0	0	0	1	VVI	tachy brady syndrome	2	0	2.25	19.3	0
05/11/1956	M	0	0	0	1	0	0	0	0	DDD	Atrial flutter with pauses	2	0	8.15	32.7	0
02/02/1965	M	0	na	na	na	na	na	na	na	DDD	High Degree AV Block	2	0	7.29	17.9	1
18/12/1943	M	0	0	0	1	0	0	0	0	DDD	High Degree AV Block	2	0	7.1	73.7	0
21/10/1936	M	1	0	0	0	0	0	0	0	DDD	sinus bradycardia	2	0	1.54	7.45	0
02/12/1939	M	1	0	1	1	1	0	0	0	DDD	complete heart block	2	0	3.54	10.7	0
22/03/1948	M	1	0	0	1	0	0	0	0	DDD	second degree heart block	2	0	6.57	52.7	0
02/08/1965	M	1	0	1	0	1	0	1	1	DDD	pauses up greater than 6 seconds	2	0	9.06	39.1	0
09/08/1937	M	0	1	0	0	0	0	0	0	VVI	tachy brady syndrome	2	0	3.06	23.4	0
03/01/1932	M	0	0	0	0	0	1	0	1	DDD	tachy brady syndrome	2	1	6.23	21.1	0
13/07/1952	M	0	1	0	1	0	0	0	0	DDD	complete heart block	2	0	7.46	38.2	0
13/08/1924	M	0	0	0	0	0	1	0	0	VVI	tachy brady syndrome	2	0	1.33	9.94	0
09/03/1946	F	0	na	na	na	na	na	na	na	DDD	Complete Heart Block	2	0	2.33	8.82	1
11/09/1955	M	0	0	0	0	0	1	0	0	DDD	trifasicular block	2	0	10.08	18	0
16/01/1931	M	0	0	0	0	0	0	0	0	DDD	trifasicular block	2	0	2.45	16	0
21/02/1936	F	0	0	0	1	0	1	0	0	DDD	tachy brady syndrome	2	0	1.53	8.85	0
22/03/1948	M	1	0	0	0	0	0	0	0	DDD	second degree heart block	2	1	3.3	17.9	0
07/07/1945	M	1	0	0	0	1	0	0	0	VVI	complete heart block	2	1	2.57	14.6	0
21/04/1935	M	1	0	0	1	0	1	0	0	DDD	complete heart block	2	4	na	na	0
14/01/1954	M	0	0	0	0	0	0	0	0	DDD	trifasicular block	2	0	na	na	0
20/04/1936	F	1	0	0	1	0	1	0	0	DDD	tachy brady syndrome	2	0	na	na	0
01/08/1969	M	0	0	0	0	0	0	0	0	DDD	pauses up greater than 6 seconds	2	0	na	na	0
23/07/1936	F	0	0	0	1	0	1	0	0	VVI	tachy brady syndrome	2	0	na	na	0
28/10/1942	F	1	0	0	1	0	0	0	0	DDD	second degree heart block	2	0	na	na	0
15/04/1959	F	0	0	0	0	0	0	0	1	DDD	complete heart block	2	0	na	na	0
23/01/1951	F	0	0	0	1	0	1	0	1	DDD	complete heart block	2	0	na	na	0
26/07/1946	M	1	0	0	0	0	0	0	0	DDD	High Degree AV Block	2	0	na	na	0
20/04/1938	M	1	0	1	0	0	0	0	0	DDD	second degree heart block	2	0	na	na	0
31/10/1928	F	0	0	1	1	1	1	0	0	DDD	complete heart block	2	0	na	na	0
19/04/1940	F	1	0	0	1	0	1	0	0	DDD	tachy brady syndrome	2	0	na	na	0
08/06/1963	F	0	0	0	0	0	0	0	0	DDD	pauses up greater than 6 seconds	2	0	na	na	0
18/05/1932	F	0	0	1	0	1	1	0	0	DDD	complete heart block	2	0	na	na	0
30/08/1931	F	1	0	0	1	0	0	0	0	VVI	tachy brady syndrome	2	0	na	na	0
26/11/1933	F	0	0	0	1	0	0	0	0	DDD	tachy brady syndrome	2	0	na	na	0
15/01/1937	M	0	0	0	1	0	0	0	1	DDD	compelte heart block	2	0	na	na	0
13/03/1950	M	1	0	0	1	0	0	0	0	DDD	trifasicular block	2	0	3.15	27	0
26/04/1930	M	0	0	0	1	0	1	0	0	VVI	trifasicular block	2	0	3.15	27	0
03/09/1955	M	0	0	0	0	0	0	0	1	DDD	complete heart block	2	0	3.12	13.4	0
05/02/1933	M	0	0	0	1	1	1	0	0	VVI	pauses up greater than 6 seconds	2	0	1.53	9.56	0
18/08/1941	M	1	0	0	0	0	0	0	0	DDD	second degree heart block	2	0	4	32.4	0
24/04/1949	F	0	0	0	0	0	0	0	0	DDD	pauses	2	0	5.24	61.5	0
26/12/1944	M	0	0	0	1	0	0	0	0	VVI	pauses	2	0	1.15	6.61	0
01/08/1954	M	0	0	0	1	0	0	0	0	DDD	second degree heart block	2	0	2.27	10.9	0
09/07/1948	M	1	0	0	0	0	0	0	0	DDD	sinus bradycardia	2	0	5.76	26.6	0
15/11/1944	F	0	0	0	0	0	0	0	0	DDD	second degree heart block	2	0	2.57	7.34	0
28/12/1947	M	1	0	0	0	0	0	0	0	DDD	second degree heart block	2	0	2.47	23.4	0
12/03/1957	M	0	0	0	0	0	0	0	0	DDD	tachy brady syndrome	2	0	4.49	71.3	0
05/09/1946	M	1	0	0	1	1	1	0	0	DDD	tachy brady syndrome	2	0	4.08	30.5	0
21/05/1928	M	0	0	0	na	na	na	na	na	DDD	trifasicular block	2	0	6.57	19.2	1
23/08/1943	F	1	0	0	1	0	0	0	0	VVI	Slow atrial fibrillation	2	0	1.52	10.2	0
14/06/1944	F	0	0	1	0	0	0	0	0	DDD	second degree heart block	2	0	9.27	39.6	0
16/03/1937	M	1	0	0	1	0	0	0	0	VVI	Slow atrial fibrillation	2	0	4.42	90.4	0
24/05/1942	M	0	0	0	0	0	0	0	0	DDD	second degree heart block	2	0	4.27	34.7	0
27/12/1940	M	1	0	0	0	1	0	0	0	VVI	AF with tachy/brady	2	0	1.58	13.9	0
19/05/1931	M	0	0	0	0	0	0	0	0	VVI	AF with TFB	2	1	1.49	11.4	0
05/05/1943	M	0	0	0	1	1	1	0	1	DDD	AF, tachy brady	2	0	11.14	61	0
12/12/1949	F	1	0	1	0	0	0	0	0	DDD	Second degree AV block	2	0	8.03	16.6	0
03/12/1950	M	0	0	0	0	0	0	0	1	DDD	Mobitz 2	2	0	3.13	5.52	0
27/11/1932	M	0	0	0	0	0	1	0	0	VVI	Slow AF	2	0	1.39	12.5	0
11/09/1932	M	1	0	1	1	1	0	0	0	DDD	Trifascicular block	2	0	11.58	49.4	0
02/04/1953	M	0	0	0	0	1	0	0	0	DDD	Sinus bradycardia	2	0	6.27	18.7	0
08/04/1955	M	0	0	0	0	0	0	0	0	DDD	tachy/brady	2	0	4.32	20.5	0
26/02/1956	F	1	0	0	0	0	0	0	0	DDD	Tachy/brady	2	0	8.46	39.8	0
07/03/1943	M	0	0	1	1	0	0	0	0	DDM	CHB	2	0	3.22	24	0
03/08/1933	M	0	0	0	0	0	1	0	0	VVI	Fall, pauses, CHB	2	0	4.32	19.1	0
24/05/1939	F	0	0	0	1	1	1	0	0	VVI	Mobitz 1 or 2	2	0	4.09	11.3	0
15/02/1967	M	1	0	0	1	0	0	0	0	DDD	10 sec pause	2	0	2.05	9.01	0
22/02/1921	F	0	0	0	1	0	0	0	1	DDD	CHB	2	0	na	na	0
21/04/1929	M	0	0	0	0	0	0	0	0	DDD	Second degree AV block	2	0	0.52	6	0
25/04/1952	M	1	0	0	1	0	0	0	0	DDD	High degree AV block	2	0	1.19	10.1	0
20/10/1946	M	0	0	1	1	1	0	0	0	DDD	Sinus bradycardia with junctional	2	0	6.28	52	0
24/10/1933	F	0	0	0	na	na	na	na	na	DDD	junctional brady,	2	0	5.56	29.6	1
17/03/1943	F	0	0	1	1	0	1	0	1	DDD	Symptomatic TFB	2	0	10.07	45.7	0
08/09/1935	M	1	0	1	1	0	0	0	1	DDD	2:1 AV blcok	2	2	4.49	25.6	0
06/05/1962	F	0	0	0	0	1	0	0	0	DDD	chb	2	0	5.24	24	0
04/08/1946	F	0	0	0	1	0	0	0	0	DDD	Tachy -brady	2	0	na	na	0
11/11/1949	M	1	0	0	1	0	0	0	0	DDD	High degree AV blcok	2	0	5.28	15.5	0
16/12/1937	M	1	0	0	1	0	0	0	0	DDD	Bifascicular block	2	0	1.58	13.3	0
30/09/1959	M	0	0	1	0	1	0	0	0	DDD	AF with high degree AV block	2	0	4.56	42.4	0
17/05/1942	M	0	0	0	1	0	0	0	1	DDD	CHB	2	0	2.28	16.7	0
24/03/1944	M	0	0	0	1	0	0	0	0	VVI	AF, trifascicular block	2	0	1.35	12.3	0
13/05/1949	M	0	0	0	1	0	0	0	0	DDD	Fall, 5.6 sec pause	2	4	3.5	30.5	0
10/02/1930	F	0	0	0	0	0	0	0	0	DDD	CHB	2	0	4.46	16	0
18/05/1962	M	1	0	0	1	1	0	0	0	DDD	AF, slow VR with pauses	2	0	2.35	na	0
24/01/1947	M	0	0	0	1	0	0	0	0	DDD	Slow AF	2	0	5.39	30	0
20/12/1933	F	1	0	1	1	0	1	0	0	DDD	Symptomatic sinus arrest	2	0	3.31	15.9	0
23/02/1962	F	0	0	0	1	0	0	0	0	DDD	complete heart block	2	0	3.03	12.5	0
12/05/1955	M	0	na	na	na	na	na	na	na	DDD	sinus bradycardia	2	0	3.44	11.2	1
22/02/1930	M	1	0	0	0	1	0	0	0	VVI	Slow atrial fibrillation	2	0	na	4.4	0
12/11/1947	M	0	0	0	0	0	0	0	0	DDD	CHB	2	0	4.4	32.3	0
07/05/1928	M	0	0	0	na	na	na	na	na	VVI	AF with slow VR	2	0	3.37	15.7	1
27/06/1943	F	0	0	0	0	0	1	0	0	DDD	Sick sinus syndrome	2	2	3.14	9.17	0
17/03/1928	M	0	0	0	1	0	1	0	1	VVI	Intermitted CHB	2	5	2	12.2	0
16/05/1935	M	1	0	0	0	0	0	0	0	VVI	AF with poor chronotrophy	2	0	2.35	19.2	0
02/01/1947	M	0	0	0	1	0	0	0	0	DDD	pauses up to seven seconds	3	0	4.18	20.9	0
04/06/1935	F	0	0	1	1	0	1	0	0	DDD	complete heart block	3	0	2.56	46.5	0
01/04/1931	F	0	0	0	1	0	0	0	0	DDD	tachy-brady syndrome	3	0	1.13	3.92	0
16/07/1935	M	1	0	0	0	0	0	0	0	DDD	AVNRT requiring ablation	3	0	3.56	21.4	0
14/08/1923	F	0	0	0	0	0	1	0	0	VVI	complete heart block	3	0	1.4	3.16	0
30/12/1942	M	0	0	0	1	0	0	0	0	DDD	complete heart block	3	0	1.4	10.5	0
22/10/1958	F	1	0	0	0	0	0	0	0	VVI	pauses up greater than 6 seconds	3	3	5.51	15.3	0
09/09/1943	F	0	0	0	1	0	0	0	0	VVI	atrial fibrillation	3	0	2.09	6.67	0
29/11/1934	M	0	0	0	0	0	0	0	0	DDD	significant bradycardia	3	0	na	na	0
21/02/1933	M	1	0	0	0	0	0	0	0	VVI	tachy brady syndrome	3	0	1.29	3.96	0
08/03/1935	M	0	0	0	0	0	0	0	0	DDD	bradycardia	3	4	na	na	0
11/09/1947	M	1	0	0	0	1	1	0	0	DDD	complete heart block	3	0	na	na	0
23/11/1950	M	0	0	0	0	0	0	0	0	DDD	complete heart block	3	0	na	na	0
08/04/1934	M	0	0	0	0	0	0	0	0	VVI	complete heart block	3	0	na	na	0
02/04/1930	F	1	0	1	1	1	1	0	0	VVI	complete heart block	3	0	na	na	0
25/01/1934	F	0	1	1	1	0	0	0	0	VVI	tachy brady syndrome	3	0	na	na	0
02/04/1928	M	0	0	0	0	0	1	0	0	VVI	trifasicular block	3	0	0.58	3.38	0
15/02/1935	M	0	0	0	0	0	0	0	0	VVI	sinus bradycardia	3	4	3.52	28.5	0
01/06/1949	M	0	0	0	0	0	0	0	0	DDD	long QT	3	0	1.38	9.31	0
05/02/1943	M	0	0	0	0	0	0	0	0	DDD	complete heart block	3	0	5.36	39.5	0
18/06/1933	F	1	0	0	1	0	0	0	0	VVI	tachy brady syndrome	3	0	1.22	8.58	0
10/07/1944	M	0	0	0	0	0	0	0	0	DDD	sinus bradycardia	3	0	1.35	10.1	0
27/10/1952	M	0	0	0	1	0	0	0	0	DDD	pauses	3	0	3.38	19.5	0
12/08/1938	M	0	0	0	na	na	na	na	na	DDD	tachy brady syndrome	3	0	1.34	7.72	1
12/02/1946	M	0	0	0	0	0	0	0	0	DDD	second degree heart block	3	0	2.12	12.4	0
28/01/1939	F	0	0	0	0	0	0	0	0	VVI	complete heart block	3	0	2.13	7.47	0
05/07/1944	F	0	0	0	na	na	na	na	na	DDD	Sinus bradycardia with junctional	3	0	1.53	4.44	1
17/07/1927	M	1	0	0	1	0	0	0	0	VVI	Tachy-brady	3	0	0.54	8.48	0
20/08/1947	M	1	0	0	1	0	0	0	0	DDD	Atrial flutter with pauses	3	0	2.2	14.3	0
18/04/1944	M	1	0	0	0	0	0	0	0	VVI	AF with slow VR	3	0	2.22	17.03	0
26/12/1949	F	0	0	0	na	na	na	na	na	VVI	CHB	3	0	2.33	12.35	1
05/03/1941	F	1	0	0	0	0	0	0	1	VVI	AF with pauses on ILR	3	0	1.18	5.16	0
